# Expansion of the neck reconstituted the shoulder–diaphragm in amniote evolution

**DOI:** 10.1111/dgd.12243

**Published:** 2015-10-29

**Authors:** Tatsuya Hirasawa, Satoko Fujimoto, Shigeru Kuratani

**Affiliations:** ^1^ Evolutionary Morphology Laboratory RIKEN 2‐2‐3 Minatojima‐minami, Chuo‐ku Kobe 650‐0047 Japan

**Keywords:** amniote evolution, congenital diaphragmatic hernia, diaphragm, lateral plate mesoderm, neck

## Abstract

The neck acquired flexibility through modifications of the head–trunk interface in vertebrate evolution. Although developmental programs for the neck musculoskeletal system have attracted the attention of evolutionary developmental biologists, how the heart, shoulder and surrounding tissues are modified during development has remained unclear. Here we show, through observation of the lateral plate mesoderm at cranial somite levels in chicken–quail chimeras, that the deep part of the lateral body wall is moved concomitant with the caudal transposition of the heart, resulting in the infolding of the expanded cervical lateral body wall into the thorax. Judging from the brachial plexus pattern, an equivalent infolding also appears to take place in mammalian and turtle embryos. In mammals, this infolding process is particularly important because it separates the diaphragm from the shoulder muscle mass. In turtles, the expansion of the cervical lateral body wall affects morphogenesis of the shoulder. Our findings highlight the cellular expansion in developing amniote necks that incidentally brought about the novel adaptive traits.

## Graphical abstract

Analyses of the lateral plate mesoderm at cranial somite levels revealed that the deep part of the lateral body wall is moved concomitant with the caudal transposition of the heart, resulting in the infolding of the expanded cervical lateral body wall into the thorax in amniote embryos. In amniote evolution, this infolding process provided a basis for the evolution of diverse shoulder structures, which deviate from those in the anamniote grade, and the diaphragm represents the most distinct example.

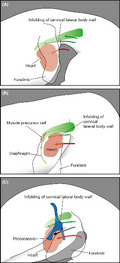

## Introduction

**Figure 1:**
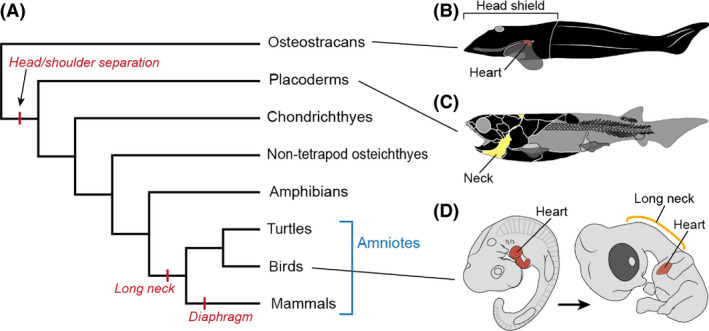
Evolution of the vertebrate neck. (A) Phylogenetic tree of gnathostomes. (B) Stem gnathostome *Norselaspis glacialis* (Osteostraci; reconstruction based on Janvier 1981). (C) Stem jawed vertebrate *Compagopiscis croucheri* (Placodermi; reconstruction based on Trinajstic *et al*. 2013; redrawn from Hirasawa & Kuratani 2015). (D) Embryonic development of the chicken, from HH stage 22 (left) to stage 30 (right).

The amniote neck is an evolutionarily novel trait that stands out among vertebrates for its extensive movability. The evolutionary acquisition of such an elongated movable neck involves modifications of the region between the head and shoulder, where the heart and gills are positioned in anamniote vertebrates (Fig. 1). Many studies have investigated the evolution of the neck muscles (e.g., Matsuoka *et al*. 2005; Theis *et al*. 2010; Kuratani 2013; Trinajstic *et al*. 2013), but the repositioning of internal organs, including the heart, in neck evolution has not been extensively considered.

According to the fossil record, osteostracans, one of the most basal vertebrate lineages that possessed pectoral fins, had a shoulder that was connected to the head at the level of the heart (Fig. 1B; Janvier 1981). The true neck first appeared in jawed vertebrates through the separation of the head and shoulder skeletons (Fig. 1C; Trinajstic *et al*. 2013), and since terrestrialization gills and aortic arch arteries have secondarily been lost in some tetrapod lineages (Romer & Parsons 1977; Schoch & Witzmann 2011). Despite these modifications of the head–trunk interface, anamniotes have not attained complete separation of the head and shoulder in terms of spinal nerve morphology (van der Horst 1934). That is, the hypoglossal nerve and brachial plexus are still juxtaposed, not intervened by cervical nerves, as seen in amniotes. Long necks evolved exclusively in the amniotes, with, for example, seven well‐specified cervical vertebrae in mammals (Narita & Kuratani 2005) and as many as 76 in the elasmosaur *Albertonectes* (Kubo *et al*. 2012).

An elongated trachea is also associated with neck elongation (Janis & Keller 2001; Perry & Sander 2004), as the internal organs are mostly encapsulated in the thoracic region in amniotes, an anatomical configuration that differs conspicuously from that in anamniotes. In particular, the heart is encapsulated in the thorax, apart from the head, in the amniote body (Fig. 1D). Thus, the development of the amniote neck occurs in concert with the amniote‐specific localization of the heart.

During embryonic development, the heart initially develops in close association with the head, and indeed the myocardium and head develop from a shared mesodermal cell population regulated by *Tbx1* (Kelly *et al*. 2004; Lescroart *et al*. 2015). These mesodermal cells, the cardiopharyngeal muscle progenitors (Diogo *et al*. 2015), do not differentiate in the post‐occipital region (Kelly 2012; Tanaka 2013). On the other hand, anteroposterior positioning of the forelimb is regulated by both *Hox4* and *Hox5* paralogues (Minguillon *et al*. 2012), that is, independently of head development. Accordingly, in long‐necked vertebrates, the heart initially develops cranial to the forelimb and later shifts caudally into the thoracic domain. This caudal shift represents a morphogenetic movement unique to the amniote heart. Because the heart is contiguous with the lateral body wall at the common cardinal vein (ductus cuvieri*)*, a developmental remodeling is expected to occur in the cervical lateral body wall during this shift. However, the mesenchymal behavior of this region has not been analyzed in detail so far.

To observe this movement in the avian embryo, we performed chicken–quail chimera experiments, in which a part of the lateral plate mesoderm at cervical somite levels (from the 7th to 17th somites) was grafted homotopically between chicken and quail embryos. Cell lineages of the somatopleure have been analyzed to elucidate their contribution to bones (Chevallier 1977), but in this study we focused on changes in the distribution of these cells during the putative lateral body wall deformation. For this purpose, we observed the distributions of transplanted cells in embryos at the stage when the musculoskeletal precursors are still surrounded by mesenchymal connective tissues, and we analyzed the relationship between the distributions of quail cells and the positions of the heart, thorax, and shoulder.

The heart passes through the base of the forelimb bud during its encapsulation into the thorax, and this movement is expected to affect the development of the shoulder. The diverse morphologies of shoulder musculoskeletal systems (Parker 1868; Romer 1924) may have arisen in such an embryonic enviroment, which results in a craniocaudal displacement of embryonic tissue. Therefore, understanding the evolution of the amniote neck is also pivotal to unraveling the developmental bases of other novel traits, in particular the mammalian diaphragm and the turtle shoulder, which show embryonic caudal transpositions of primordial structures that initially develop at the neck–shoulder boundary.

During mammalian embryonic development, the precursor of the diaphragm, an evolutionary novelty in the lineage leading to mammals, arises adjacent to those of the forelimb muscles (Babiuk *et al*. 2003). We previously proposed that the diaphragm was acquired through a partial duplication of shoulder muscle precursors, in particular the subscapular muscle (Hirasawa & Kuratani 2013). In this scenario, addition and fixation of cervical vertebral counts in the lineage leading to mammals were evolutionarily linked to the individualization of the novelty, the diaphragm. Although this hypothesis has not yet been proven experimentally, recent studies have provided supporting evidence of the differentiation of motor neuron identities (Machado *et al*. 2014; Jung & Dasen 2015).

The shoulder skeleton of the turtles exhibits an evolutionary aberration with respect to its position and morphology. In turtle development, the primordium of the shoulder skeleton becomes folded beneath the ribs, that is, beneath the carapace precursor (Nagashima *et al*. 2009). In the turtle body, the procoracoid element, homologous to the cranial coracoid in stem amniotes, extends caudomedially (Nagashima *et al*. 2013), suggesting a predominant craniocaudal displacement of the shoulder structure during evolution. From this perspective, we also investigated the relationships between these caudal transpositions of the shoulder–diaphragm structure and the caudal shift of the heart, by observing deformations of spinal nerve axons as a proxy.

## Materials and methods

### Lineage tracing of lateral body wall cells at the neck level in birds

Fertilized eggs of chicken (*Gallus gallus*) and Japanese quail (*Coturnix coturnix*) were purchased from local farms and incubated at 38°C under moist conditions.

**Figure 2:**
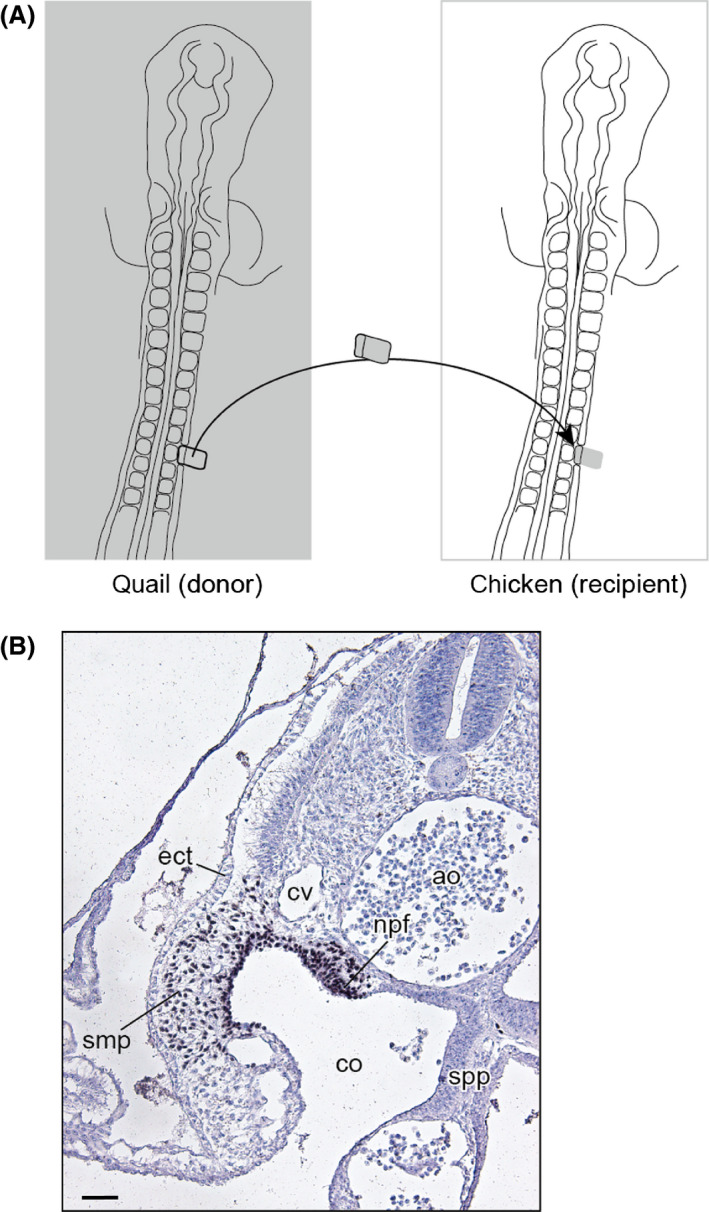
Experimental procedure of the chicken–quail chimera. (A) Schematic drawing of the transplantation. (B) Transverse section of an operated (at the 10th somite level) embryo collected 16 h after the operation, with quail cell marker antibody (QCPN)‐immunohistochemical and hematoxylin staining. ao, aorta; co, coelom; cv, cardinal vein; ect, ectoderm; npf, nephric fold; smp, somatopleure; spp, splanchnopleure. Scale bar equals 50 μm.

At Hamburger and Hamilton (HH) stages 11 and 12 (Hamburger & Hamilton 1951), the right somatopleural and intermediate mesoderms adjacent to one somite of a chicken embryo (recipient) were excised *in ovo* using an electrolytically sharpened tungsten needle with the aid of 1 μL dispase solution (dispase II [Wako Pure Chemical Industries, Osaka, Japan], at a dilution of 500 protease units/mL). At the same stages, the right somatopleural and intermediate mesoderms at the same level of a quail embryo (donor) were excised *ex ovo* and transplanted homotopically into the recipient chicken embryo (Fig. 2).

The manipulated chicken embryos were then fixed with Serra's fixative (Serra 1946) at later developmental stages. The obtained samples were embedded in paraffin and sectioned either transversely or sagittally at a thickness of 8 μm. The distributions of transplanted quail cells were observed with the aid of immunohistochemistry (quail cell marker antibody [QCPN; Developmental Studies Hybridoma Bank http://dshb.biology.uiowa.edu/], at a dilution of 1:2). To enhance the immunoreaction signal, avidin‐biotinylated peroxidase complex sensitization (Vectastain Elite ABC Standard Kit VEC PK‐6100; Vector Laboratories, Burlingame, CA, USA) was performed following the manufacturer's instructions. After immunostaining, the histological sections were stained with Alcian blue and hematoxylin.

### Observation of mouse and turtle embryos

Mouse (*Mus musculus*, C57BL/6 strain) embryos were collected in accordance with the guidelines of our Institutional Animal Ethics Committee (approval: AH21‐08). The embryos were staged according to Kauffman (1992).

Chinese soft‐shelled turtle (*Pelodiscus sinensis*) eggs were purchased from a local farm and incubated at 30°C. The embryos were staged according to the Tokita and Kuratani (TK) system (Tokita & Kuratani 2001).

The embryos were fixed with Serra's fixative. Histological sections were stained with Alcian blue, hematoxylin, and eosin, as well as immunohistochemically (anti‐acetylated tubulin antibody, T6793 [Sigma‐Aldrich, St Louis, MO, USA], at a dilution of 1:400).

### Imaging

Serial histological sections were digitized with a DP70 camera (Olympus Corp., Tokyo, Japan) mounted on a BX60 microscope (Olympus Corp.). The digitized images of serial sections were aligned and reconstructed three‐dimensionally, using the Avizo software (FEI Visualization Sciences Group, Hillsboro, OR, USA). Each embryonic component was labeled on the sections and subsequently combined to generate a three‐dimensional model.

## Results

### Somatopleure at the neck level shifts caudally into the thorax in the avian embryo

In the first series of experiments, the quail somatopleure at the cervical somite level was grafted homotopically into chicken embryos at HH stages 11 and 12. Nine of the 15 chimeric embryos were harvested 5 days after grafting, at a stage corresponding to HH stage 30 (Table 1). Five chimeric embryos had died before HH stage 30, and one was excluded from the analysis because it had a scar on the operated side. Among the nine evaluated specimens, eight were used for detailed histological observation. In one evaluated specimen, no QCPN signal was detected.

**Table 1:** Experimental data for the chicken–quail chimeras

Level of the grafted somatopleure†	Operated stage (total number of somites)	Figure
7th somite	HH stage 11 (14)	Fig. 4A,B
8th somite	HH stage 11 (14)	
8th somite	HH stage 12 (18)	Fig. 3
10th somite‡	HH stage 11 (15)	
11th somite (*n* = 2)	HH stage 12 (16)	Fig. 4C,D
12th somite	HH stage 12 (15)	
12th somite	HH stage 12 (16)	
14th somite	HH stage 12 (17)	Fig. 4E,F

^†^Only the chimeric embryos showing apparently normal development at HH stage 30 are listed. ^‡^No quail cell marker antibody (QCPN) signal was detected.

**Figure 3:**
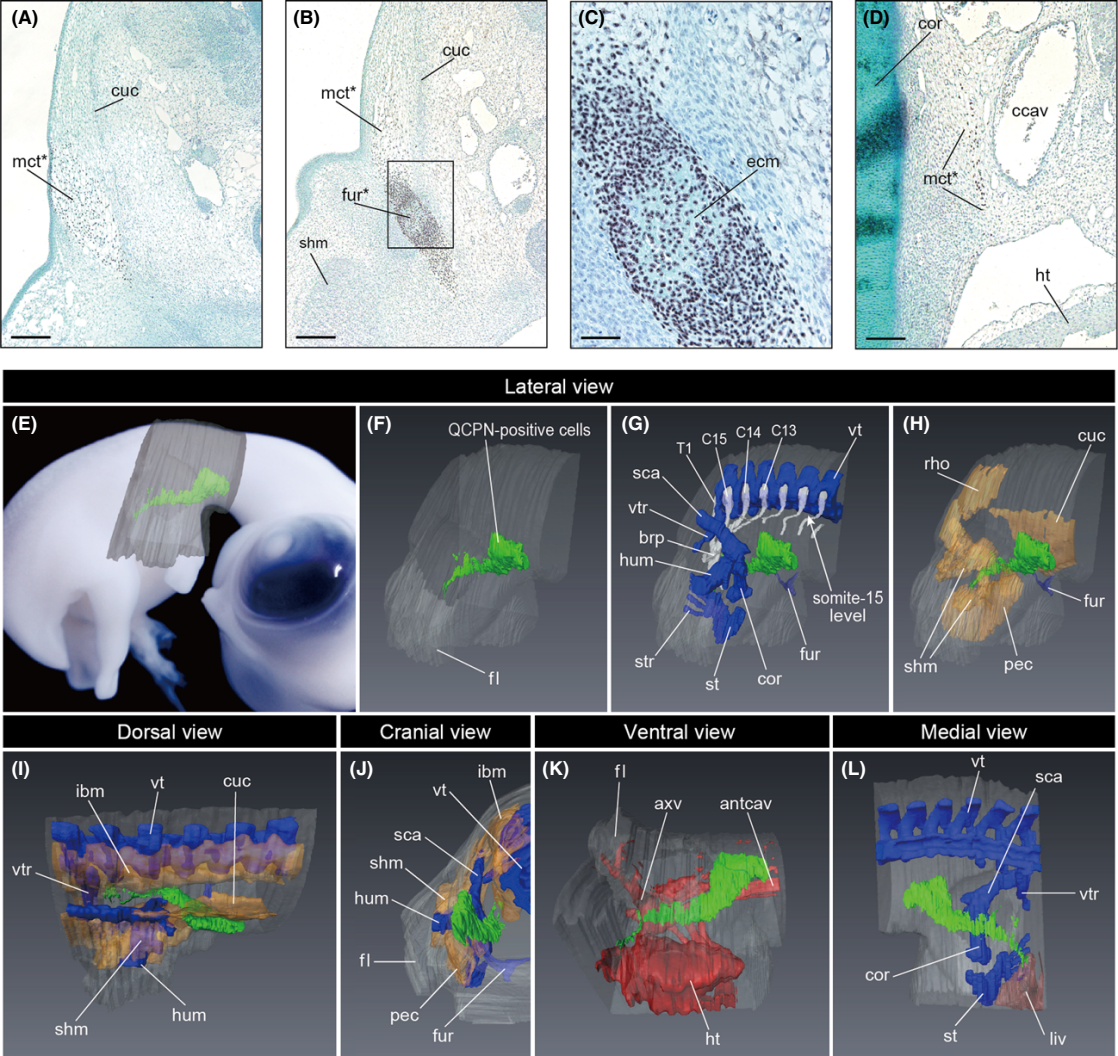
Distribution of the cells derived from the somatopleure at the eighth somite level in the chicken embryo. The quail‐to‐chicken transplantation of the somatopleure at the eighth somite level was performed at HH stage 12, and the embryo was fixed at HH stage 30. (A–D) Transverse sections with quail cell marker antibody (QCPN)‐immunohistochemical, hematoxylin, and Alcian blue staining at the neck level (A), rostral shoulder level (B, and its higher magnified image in C), and caudal shoulder level (D). ccav, common cardinal vein; cor, coracoid; cuc, cucullaris muscle; ecm, Alcian blue–stained extracellular matrix; fur, furcula; ht, heart; mct, mesenchymal connective tissue; shm, shoulder muscles. Asterisk indicates QCPN‐positive cells. Scale bars equal 200 μm in A and B, 50 μm in C, and 100 μm in D. (E–L) Distribution of the QCPN‐positive cells in a three‐dimensional model. (E) Position of the three‐dimensional model in lateral view. (F) Contour of the embryo and QCPN‐positive cells in lateral view. (G) Cartilages, spinal nerves and QCPN‐positive cells in lateral view. (H) Muscles and QCPN‐positive cells in lateral view. The axial muscles are not shown; the furcula is shown. (I, J) Muscles, cartilages, and QCPN‐positive cells in dorsal view (I) and cranial view (J). The rhomboid muscle is not shown in I, and the cucullaris muscle is not shown in J. (K) Heart, blood vessels, and QCPN‐positive cells in ventral view. (L) Liver, cartilage, and QCPN‐positive cells in medial view. QCPN‐positive cells, cartilage, spinal nerve, muscle, heart and blood vessels, and liver are shown in green, blue, white, yellow, red, and brown, respectively. antcav, anterior cardinal vein; axv, axillary vein; brp, brachial plexus; C13–C15 and T1, cervical spinal nerve 13–15 and thoracic spinal nerve 1; cor, coracoid; cuc, cucullaris muscle; fl, forelimb; fur, furcula; ht, heart; hum, humerus; ibm, intrinsic back muscles; pec, pectoralis muscle; liv, liver; rho, rhomboid muscle; sca, scapula; shm, shoulder muscles; st, sternum; str, sternal rib; vt, vertebra; vtr, vertebral rib.

Figure 3 shows the result of a case in which the quail somatopleure at the eighth somite level was grafted homotopically into a chicken host. In this chimera, transplanted somatopleure cells developed into a mesenchymal connective tissue and the furcula (Fig. 3A–D). Cranial to the furcula, QCPN‐positive cells were found in the mesenchymal connective tissue lateral to the cucullaris muscle (Fig. 3A,E–J), but not in the connective tissue of the muscle (Fig. 3A,B). Caudal to the furcula, the QCPN‐positive cells were found exclusively in the mesenchyme medial to the shoulder girdle (Fig. 3D,E–J), brachial plexus (Fig. 3G) and thoracic ribs (Fig. 3G,I). The QCPN‐positive cells were distributed caudally within the thorax, reaching the gap between the heart and liver (Fig. 3K,L).

**Figure 4:**
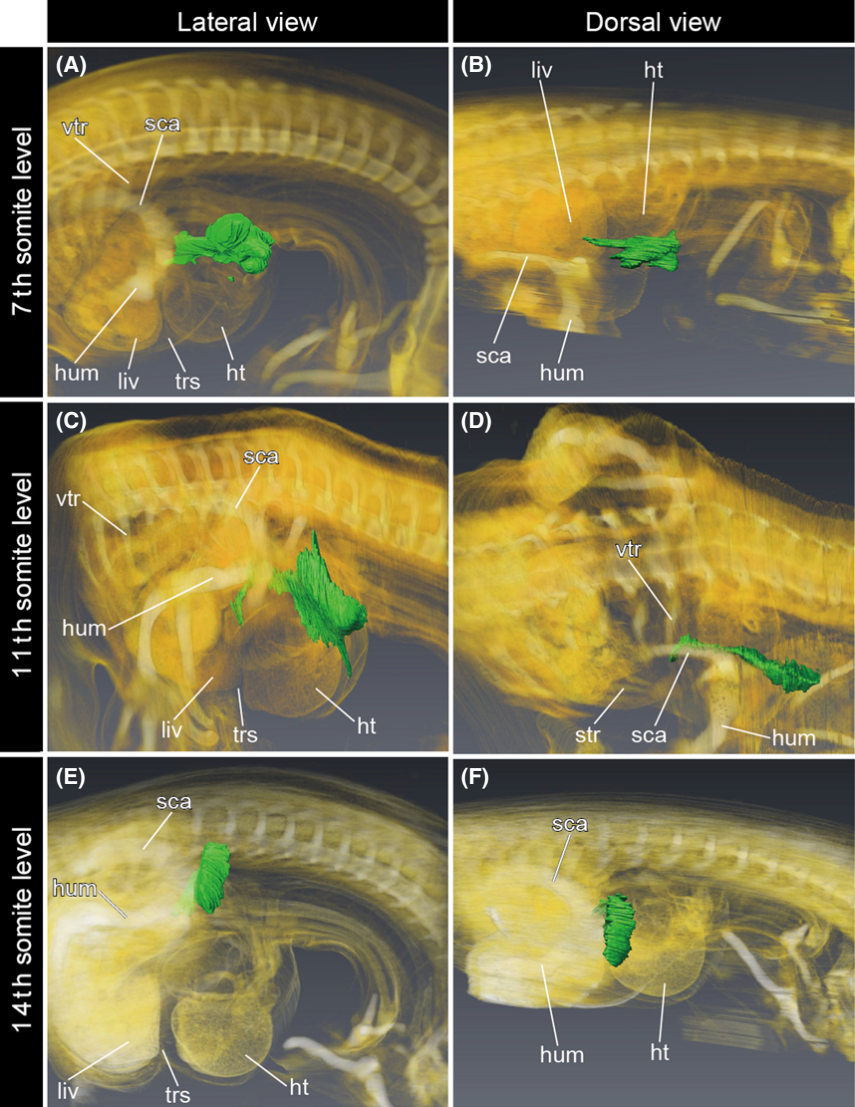
Distribution of the cells derived from the somatopleure at the cervical somite level in the chicken embryos. Three‐dimensional models were obtained by volume rendering from gray‐scale images of histological sections. Quail cell marker antibody (QCPN)‐positive cells are shown in green. (A, B) The quail‐to‐chicken transplantation of the somatopleure at the seventh somite level was performed at HH stage 11, and the embryo was fixed at HH stage 30. (C, D) The quail‐to‐chicken transplantation of the somatopleure at the 11th somite level was performed at HH stage 12, and the embryo was fixed at HH stage 30. (E, F) The quail‐to‐chicken transplantation of the somatopleure at the 14th somite level was performed at HH stage 12, and the embryo was fixed at HH stage 30. ht, heart; hum, humerus; liv, liver; rho, rhomboid muscle; sca, scapula; str, sternal rib; trs, transverse septum; vtr, vertebral rib.

Cells derived from the other part of the cervical somatopleure (Table 1) were also found medial to the shoulder and thorax (Fig. 4). In each transplantation, QCPN‐positive cells were distributed lateral to the cucullaris at the cervical level and medial to the shoulder girdle and to the thoracic ribs at the thoracic level. At the neck–shoulder boundary, QCPN‐positive cells participated in the formation of the furcula, whereas no contribution to the other bones and muscles was detected. The caudal end of the QCPN‐positive cell population always extended to the gap between the heart and liver. These results demonstrate that the somatopleure in chicken embryos at the cervical somite levels grows obliquely to the sagittal plane and is distributed within the thorax through the superior thoracic aperture.

### Phrenic nerve extends caudally in association with caudal transposition of the heart in the mammalian embryo

**Figure 5:**
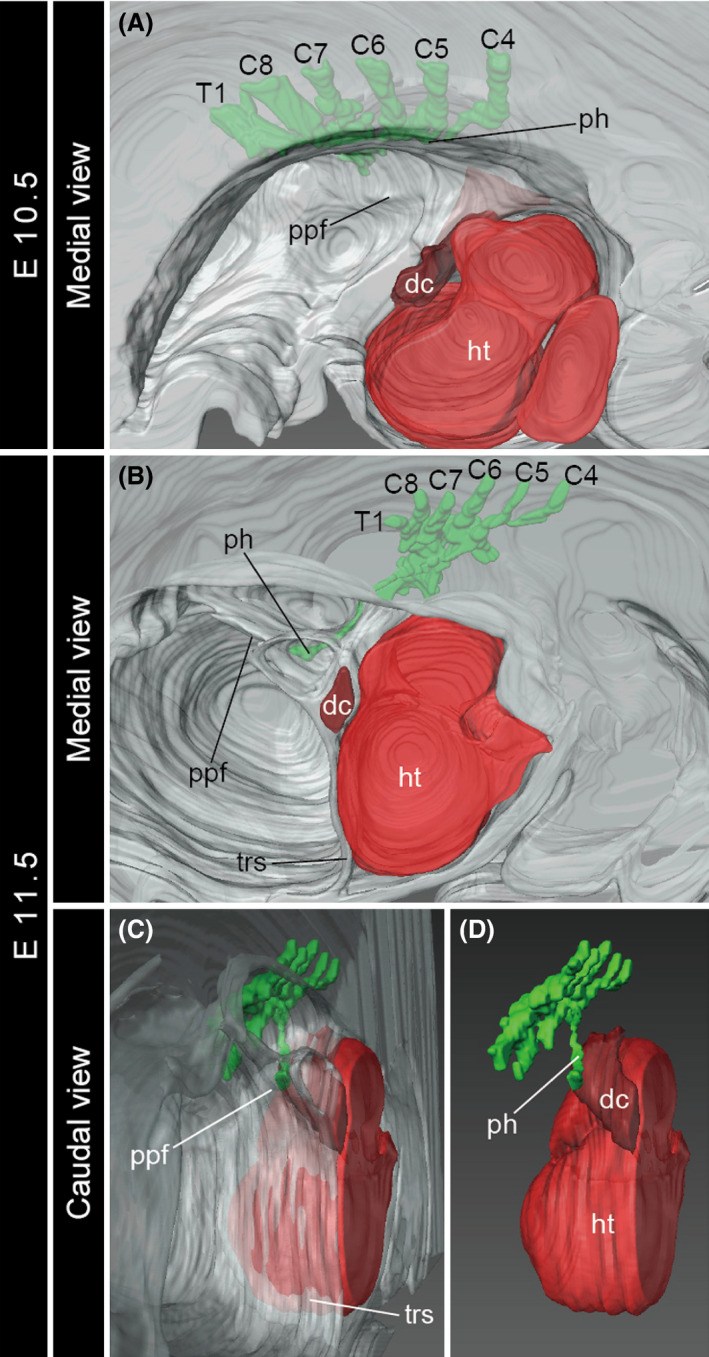
Development of the brachial plexus in the mouse embryos. Three‐dimensional models of the left half of the embryos at E10.5 (A, in medial view) and E11.5 (B, medial view; C, D, caudal view). The brachial plexus and the heart are shown in green and red, respectively. C4–C8 and T1, cervical spinal nerve 4–8 and thoracic spinal nerve 1; dc, ductus cuvieri; ht, heart; ph, phrenic nerve, ppf, pleuroperitoneal fold; trs, transverse septum.

In the mouse embryo at E10.5, the fourth cervical to first thoracic (C4–T1) spinal nerves formed the brachial plexus at the level caudal to the heart (Fig. 5A). The pleuroperitoneal fold protruded medially from the lateral body wall near the level of the sixth spinal nerve (Fig. 5A). At this stage, the primordial phrenic nerve axon and the pleuroperitoneal fold were located caudal to the ductus cuvieri, or the junction between the heart and the lateral body wall.

At E11.5, the position of the heart had shifted caudally relative to the brachial plexus, whereas the positional relationship between the distal end of the phrenic nerve and the ductus cuvieri was maintained (Fig. 5B–D). By this stage, the pleuroperitoneal fold attached to the transverse septum at the level caudal to the ductus cuvieri (Fig. 5C). Thus, a part of the cervical lateral body wall, where the phrenic nerve extends inside, later shifted caudally within the thorax.

### Brachial plexus rotates caudoventrally in association with caudal transposition of the heart in the turtle embryo

**Figure 6:**
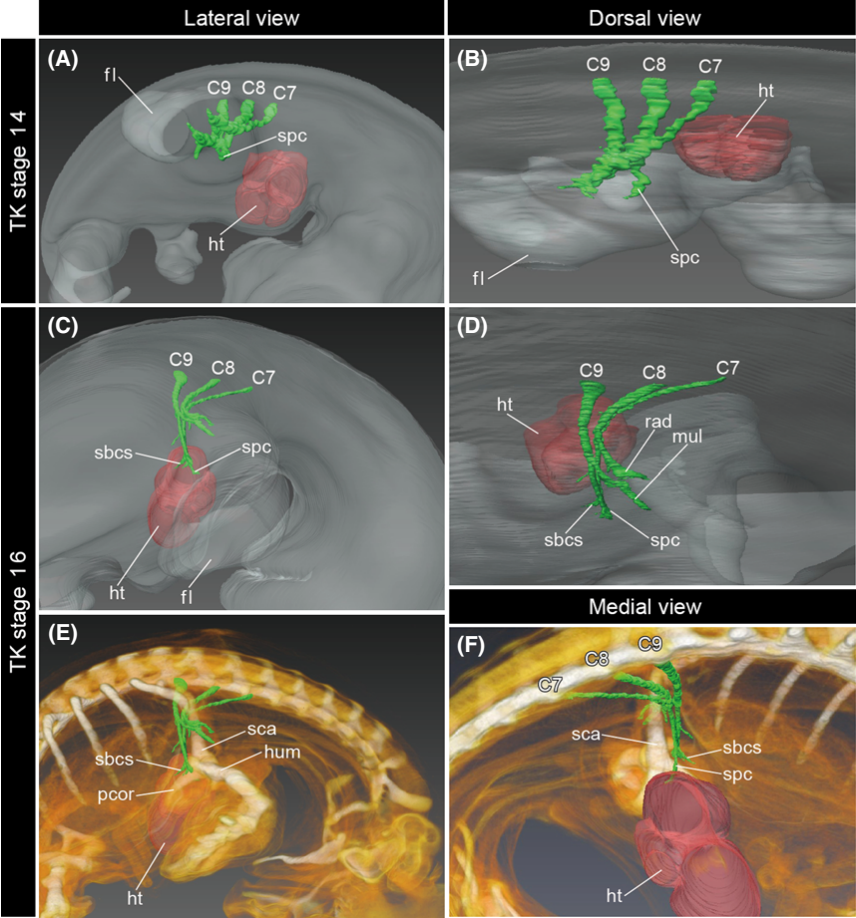
Development of the brachial plexus in the turtle embryos. Three‐dimensional models of the right half of the embryos at TK stage 14 (A, B, lateral and dorsal views) and stage 16 (C–F, lateral, dorsal, and medial views). The brachial plexus and heart are shown in green and red, respectively. C7–C9, cervical spinal nerves 7–9; fl, forelimb; ht, heart; hum, humerus; mul, medioulnar nerve; pcor, procoracoid; rad, radial nerve; sbcs, subcoracoscapular nerve; sca, scapula; spc, supracoracoid nerve.

At TK stage 14 of *P. sinensis* development, roughly corresponding to HH stage 26 of the chicken (Nagashima *et al*. 2005), the heart was still located at the cervical level (Fig. 6A,B). The seventh to ninth cervical (C7–C9) spinal nerves formed the brachial plexus at the level caudal to the heart. The supracoracoid nerve extended laterally from the cranial (or radial) part of the brachial plexus.

By TK stage 16, the heart had shifted caudally relative to the forelimb (Fig. 6C–F). At this stage, the supracoracoid nerve extended ventrolaterally, and the subcoracoscapular nerve that branches off from the radial part of the brachial plexus extended caudoventrally; a component of the brachial plexus including the supracoracoid and subcoracoscapular nerve branches was oriented in a different direction than the radial nerve, which extended laterally into the forelimb (Fig. 6D). The distal ends of this component of the brachial plexus were located at the level of the caudal side of the heart (Fig. 6F). Because the branching order of the brachial plexus is unchanged after its formation, the caudoventral torsion of a radial component of the brachial plexus indicates that the cervical lateral body wall, where the supracoracoid and subcoracoscapular nerves extend inside, shifts its position caudally from TK stages 14 to 16.

## Discussion

In the present study, we focused on changes in the positional relationship between the heart and lateral body wall during embryonic development. Because the displacements occur as changes in relative positions of embryonic anlagen, no *a priori* landmarks are available to describe the movements. For the sake of brevity, we use axial (i.e., spinal) levels as references for the directions of movements.

**Figure 7:**
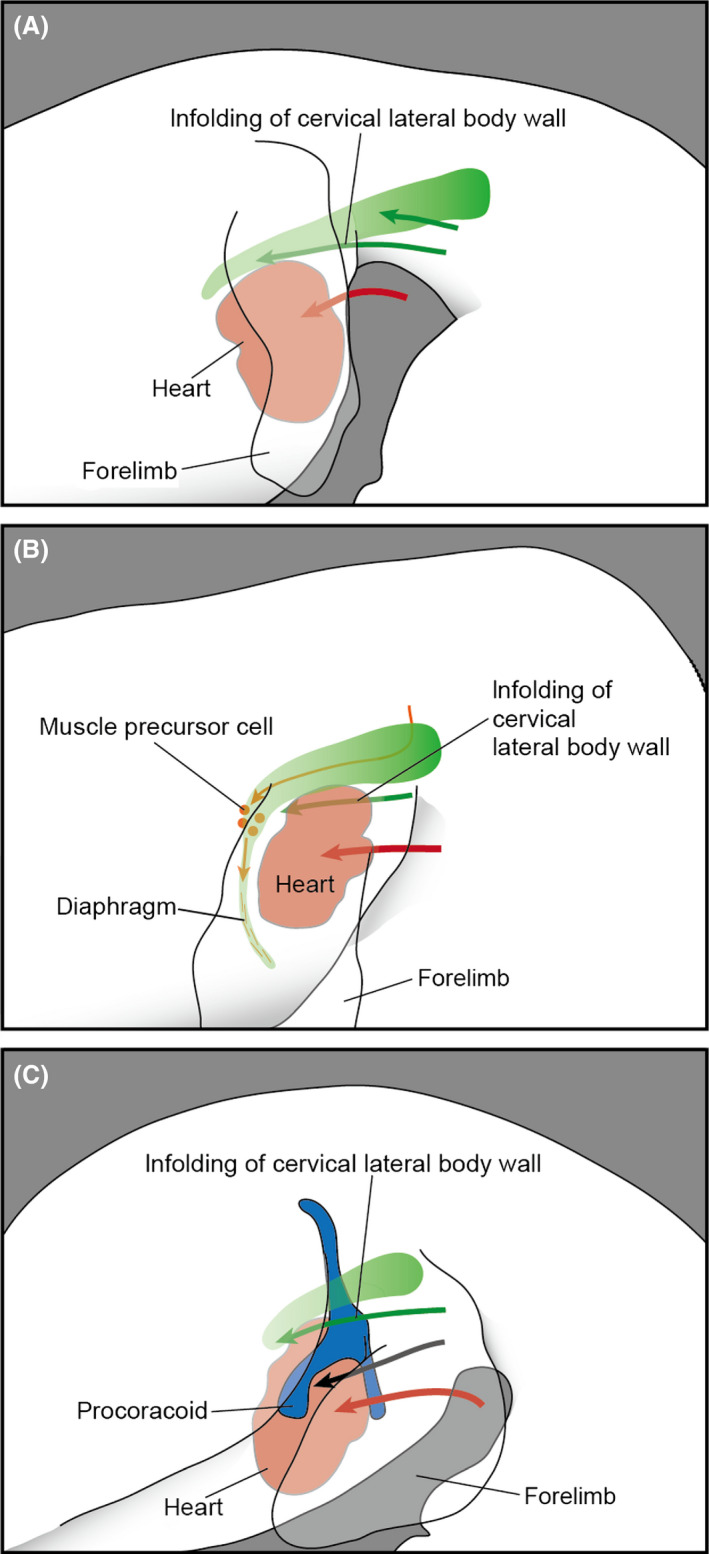
Formation of the neck in amniote embryos. The cervical lateral body wall (green) is moved caudally (green arrow) concomitant with the caudal transposition (red arrow) of the heart (red). (A) Chicken. (B) Mouse, with muscle precursor cells derived from the level cranial to the shoulder shown in orange. (C) Turtle, with the shoulder skeleton shown in blue, and the caudal transposition of the procoracoid indicated by a black arrow.

Based on our observation of the avian chimeric embryos (Figs 3, 4), it appears that the transplanted somatopleure was moved concomitant with the caudal transposition of the heart during neck formation (Fig. 7A). The cell population derived from the cervical somatopleure was distributed both lateral and medial to the musculoskeletal system (Fig. 3), which we hereafter designate as the “superficial” and “deep” parts of the cell population, respectively. The superficial part was found in the mesenchymal connective tissue in the neck region, whereas the deep part shifted caudally and disappeared from the neck region. The boundary between the superficial and deep parts always corresponded to the furcula. The deep part of the expanded cervical lateral body wall was distributed medial to the brachial plexus (Fig. 3G), suggesting that the caudal shift of the deep part did not affect the patterning of the brachial plexus in the avian embryos. By observing chimeras at HH stage 30, we found that the musculoskeletal systems and visceral organs were already well differentiated by that stage. Thus, at later stages the mesenchymal connective tissue in the deep part develops largely into the loose connective tissue lying between muscles and viscera, whereas that in the superficial part develops into the integumentary system. In other words, during avian development the cervical lateral body wall grows caudally into the thorax via the superior thoracic aperture, but does not contribute to any specific structures within the thorax.

The caudal transposition of the heart also occurs in other amniote embryos. In the mouse and turtle embryos, we observed that the shape of the brachial plexus became distorted concomitant with the positional change of the heart (Figs 5, 6). In particular, the cranial part of the brachial plexus, which branches off the phrenic nerve in the mouse and off the supracoracoid and subcoracoscapular nerves in the turtle, underwent a major deformation to bring its peripheral end caudally, while maintaining its positional relationship to the caudal side of the heart. We interpret this phenomenon as associated with the caudal transposition of the heart during neck formation (Fig. 7B,C). Specifically, the deformation of the brachial plexus is likely driven by the infolding of the expanded cervical lateral body wall into the thorax, which was observed in the chicken embryos.

The caudal transposition of the diaphragm in mammalian embryos was described previously (Mall 1910; McCrady 1938; Allan & Greer 1997; Greer *et al*. 1999). We observed this process three‐dimensionally from the beginning of phrenic nerve formation. Initially, a medial protrusion of the lateral body wall, the pleuroperitoneal fold, became distinct at the cervical level (approximately at the level of the sixth spinal nerve, Fig. 5A), and this structure and the peripheral end of the phrenic nerve shifted caudally within the thorax, in accordance with the caudal transposition of the heart (Fig. 5B). As a result, the phrenic nerve extended caudomedially within the thorax via the superior thoracic aperture. This orientation and the positional relationship with the heart are comparable to those of the expanded cervical lateral body wall observed in the chicken, suggesting that the cervical lateral body wall is moved concomitant with the caudal transposition of the heart in the mouse as well (Fig. 7B). As the non‐muscular component of the diaphragm is derived solely from the pleuroperitoneal fold (Merrell *et al*. 2015), this infolding process (Fig. 7B) likely plays a pivotal role in the proper positioning of the diaphragm. The muscle precursor cells have already migrated into the pleuroperitoneal fold before the caudal transposition of the heart (E10.5; Dietrich *et al*. 1999), thus allowing them to later differentiate within the pleuroperitoneal fold at the proper position. This passive positional shift of the muscle precursors of the diaphragm is another example of the musculoskeletal development being affected by morphogenetic movements of embryonic bodies (Lours‐Calet *et al*. 2014).

Our observations suggest that the key feature in the development of the diaphragm can be attributed to the early distribution of the muscle precursor cells in the lateral body wall, rather than the caudal shift of the primordium, which is not a mammalian‐specific process. Protrusion of the pleuroperitoneal fold is not mammalian‐specific either, because various non‐muscular coelomic septa evolved repeatedly in amniote taxa (Goodrich 1930; Duncker 1979; Klein & Owerkowicz 2006; Perry *et al*. 2010). Nevertheless, the pleuroperitoneal fold holds the developmental genetic basis behind the migration of the muscle precursor cells, which may be, at least partly, mammalian‐specific. Although the pleuroperitoneal fold initially develops at the base of the forelimb bud (Fig. 5A), the pleuroperitoneal fold is marked by a transcriptional pathway involving *Gata4*, which plays an indispensable role in the migration of muscle precursor cells in the pleuroperitoneal fold (Clugston *et al*. 2008; Longoni *et al*. 2014; Merrell *et al*. 2015). On the other hand, *Gata4* and *Gata6* may redundantly contribute to the antero‐posterior patterning in the forelimb bud (Kozhemyakina *et al*. 2014), suggesting a distinct *Gata4* function in the pleuroperitoneal fold. In our previous study (Hirasawa & Kuratani 2013), we inferred that the evolutionary origin of this mammalian‐specific embryonic environment involving *Gata4* preceded and led to a partial duplication of the precursor cells of shoulder muscles. Further analysis of the genetic basis of the muscle precursor cell migration at the base of the forelimb bud will improve our understanding of the evolutionary process leading to the diaphragm.

In the turtle embryo, the infolding of the shoulder results in an aberrant positioning of the shoulder skeleton ventral to the thoracic ribs (Nagashima *et al*. 2009). This morphogenetic movement is induced by the craniolateral growth of the turtle‐specific protrusion at the paraxial part, the carapacial ridge. The thoracic ribs of the turtle develop exclusively within the paraxial part of the embryonic body (Nagashima *et al*. 2007), and the deformation of the lateral body wall along with the growth of the carapacial ridge results in the ventral position of the shoulder against the thoracic ribs (Nagashima *et al*. 2009). In the present study, we observed that the heart shifted its position caudally during the simultaneous folding of the shoulder structure (Fig. 6). Along with the cardiac transposition, the brachial plexus rotated caudoventrally (Fig. 6C–F). Because the positional relationship between the radial part of the brachial plexus and the caudal side of the heart was maintained throughout the folding process, an infolding of the cervical lateral body wall likely involves this morphogenetic movement of the brachial plexus (Fig. 7C). Therefore, in addition to the craniolateral growth of the carapacial ridge, the caudal transposition of the heart is a key morphogenetic movement needed to rotate the shoulder.

In this study, we observed the infolding of the cervical lateral body wall into the thorax during the cardiac transposition in three amniote taxa. This morphogenetic movement, therefore, likely evolved in the common ancestor of the crown amniotes, together with the encapsulation of the heart into the thorax (Fig. 1). Furthermore, in some amniote lineages, the musculoskeletal system was reconstituted in connection with this infolding: the diaphragm primordium is separated from the shoulder in the mammalian lineage, and the shoulder structure is rotated caudoventrally in the turtle lineage. Comparable developmental remodeling may have been involved in other taxon‐specific morphologies of shoulder structures in the amniotes. For example, in Mesozoic marine plesiosaurs the clavicular girdle was medial to the shoulder girdle (Nicholls & Russell 1991), unlike those of other vertebrates (Hirasawa & Kuratani 2015). This deviated positional relationship of shoulder skeletal elements in plesiosaurs is reminiscent of the infolding of the cervical lateral body wall into the thorax: the clavicular elements were moved caudally. In amniote evolution, the caudal cellular expansion of the cervical lateral body wall during the caudal shift of the heart in the developing neck provided a basis for the evolution of diverse shoulder structures, which deviate from those in the anamniote grade, and the diaphragm represents the most distinct example.

## Author contributions

T.H. designed the study. T.H. and S.F. conducted the experiments. T.H., S.F. and S.K. wrote the manuscript.
